# β-Asarone Inhibits Amyloid-β by Promoting Autophagy in a Cell Model of Alzheimer's Disease

**DOI:** 10.3389/fphar.2019.01529

**Published:** 2020-01-17

**Authors:** Nanbu Wang, Haoyu Wang, Lingyu Li, Yunchuan Li, Ronghua Zhang

**Affiliations:** ^1^ The First Affiliated Hospital, Jinan University, Guangzhou, China; ^2^ College of Traditional Chinese Medicine, Jinan University, Guangzhou, China; ^3^ College of Pharmacy, Jinan University, Guangzhou, China

**Keywords:** β-asarone, amyloid β, Alzheimer's disease(AD), PC12, autophagy

## Abstract

Alzheimer's disease (AD) is one of the most common types of dementia that causes memory, thinking, and behavior problems. The most important feature of AD is the gradual irreversible loss of cognitive ability through the formation of amyloid β (Aβ) plaques and neurofibrillary tangles composed of tau protein. The metabolism of Aβ and tau proteins is closely related to and is affected by autophagy. Current research speculates that autophagy dysfunction leads to an increase in harmful proteins in AD. β-Asarone is the main constituent of *Acorus tatarinowii* Schott and has important effects on the central nervous system. In this paper, we primarily explored the effects of β-asarone on the clearance of noxious proteins and the associated potential mechanisms *via* autophagy in a PC12 cell AD model. A CCK-8 assay and LDH experiments were used to assess cell viability/toxicity, and SPiDER-βGal was used to detect cellular senescence. The important proteins associated with the pathogenesis of AD including APP, PS1, Aβ, BACE1, and SYN1 were analyzed by immunofluorescence (IF) and Western blot analysis. Antimycin A (A3) and cyclosporine A (CSA) were selected as the activators and inhibitors of autophagy, respectively. LC3, BECN, P62, PINK1, and Parkin protein expression were also examined by IF and Western blot analysis. The data showed that β-asarone administration significantly dose-dependently increased cell proliferation and decreased cytotoxicity; moreover, β-asarone inhibited SA-βGal and improved cell senescence. The results further showed that, compared to the model, APP, PS1, Aβ, BACE1, and p62 were reduced, while SYN1, BECN1, and LC3 were increased after treatment with β-asarone. The results of Canonical Correlation Analysis (CCA) showed a highly significant relationship between the pathological factors of AD and the protein expression of autophagy. In conclusion, our study demonstrated that β-asarone can inhibit Aβ, and this effect may occur by promoting autophagy in a cell model of AD.

## Introduction

Alzheimer's disease (AD) is the most frequent cause of dementia, and this disease is gradually escalating into a global epidemic among older adults. The major neuropathological features of AD are widely described: amyloid β (Aβ) plaques and neurofibrillary tangles, but no treatment has changed its progression (Loera-Valencia et al., 2019; Reddy et al., 2019). Apart from these clear pathological lesions, the brain's immune response also undergoes dramatic changes, including dramatic changes in microglia and astrocyte phenotypes (Henstridge et al., 2019). Although research on the pathogenesis of AD continues to be controversial, the Aβ peptide is considered to be a central player (Vassa, 2004). The drugs that have successfully completed clinical trials and are approved for the clinical treatment of AD are all based on the neuroprotective hypothesis, that is, they are all agonists or antagonists of neurotransmitter production or neurotransmitter receptors, they are based on the drugs developed by other hypotheses and are still in the stages of basic research and early clinical trials, and their prospects are still unclear (Tricco et al., 2018; von Arnim et al., 2019). Donepezil (Aricept) is a centrally acting reversible acetyl cholinesterase (AChE) inhibitor which could increase the content of ACh and improving the cognitive function of patients with AD (Reardon, 2018).

Autophagy dysfunction may lead to an increase in harmful proteins in the AD brain (Steele and Gandy, 2013; Uddin et al., 2018). Studies have reported that in AD, the maturation of autophagosomes and their retrograde pathways to neuronal bodies are impeded (Nixon, 2007). Mitophagy is the selective degradation of mitochondria by autophagy. Studies have shown that Aβ inhibits mitophagy while mitophagy induction reduces Aβ in AD *Caenorhabditis elegans*, mice, and humans (Palikaras et al., 2015; Fang et al., 2019).

On the other hand, *Acorus tatarinowii* Schott (ATS) is a commonly used herbal medicine. Previous studies have shown that this herb has positive effects on neurodegenerative diseases, such as Parkinson's disease and AD, hypoxic-ischemic encephalopathy, and cerebrovascular diseases (Fang et al., 2003; Li et al., 2010). During the previous study, researchers found the main component of ATS volatile oil is β-asarone, followed by α-asarone and γ-asarone, as determined by the total ion current (TIC). β-Asarone (cis-2,4,5-tri-methoxy-1-allyl phenyl) is the main constituent of ATS and plays an important role in the central nervous system (Deng et al., 2016; Ning et al., 2019). Our previous study showed that β-asarone may help cancer treatment by promoting temozlomide's entry into glioma U251 cells (Wang et al., 2017). At the same time, it can affect autophagy for the therapy of antitumor and lead the drug through the blood–brain barrier (BBB) or the membrane (Wang et al., 2018). Here, we report preliminary data showing that β-asarone can protect PC12 cells against Aβ_42_ induced injury. At the same time, we used antimycin A (A3) as the autophagy activator and cyclosporine A (CSA) and 3-methyladenine (3MA) as autophagy inhibitors. Here we propose that β-asarone could protect a PC12 cell model against Aβ_1-42_ damage, and this process should occur by promoting autophagy.

## Materials and Methods

### Reagents

The Aβ_1-42_ used in these experiments was acquired from Life Technologies (USA); β-asarone was purchased from NIFDC (Beijing, China), and the purity value is 96.8%; donepezil was obtained from the First Affiliated Hospital of Jinan University (Guangzhou, China); A3 was purchased from Santa Cruz Biotechnology (Santa Cruz, CA, USA); CSA was purchased from Selleck (Houston, Texas, USA); 3MA, high-glucose DMEM, FBS, trypsin, and PBS were obtained from Gibco (Gaithersburg, MD, USA); Cell Counting Kit-8 (CCK-8), Cytotoxicity LDH Assay Kit—WST (LDH), and Cellular Senescence Detection Kit—SPiDER-βGal were obtained from Dojindo Molecular Technologies, Inc. (Tokyo, Japan); the bicinchoninic acid (BCA) protein assay kit, Immunol Fluorescence Staining Kit, and Immunohistochemistry Staining Kit were obtained from Beyotime (Shanghai, China); anti-SQSTM1/P62, anti-BECN, anti-LC3 II, anti-beta amyloid, anti-BACE1, anti-synapsin1, anti-Parkin, anti-Pink1, anti-APPL, and anti-PS1 were obtained from Abcam (Cambridge, UK).

### Cell Culture and Handing

Highly differentiated PC12 cell was purchased from Shanghai BCB (TCR9). The cells were maintained in DMEM containing 10% FBS and 1% penicillin/streptomycin at 37°C in a humidified 5% CO_2_ atmosphere.

### Preparation of Oligomerization Aβ_1-42_

Aβ_1-42_ is the component found in amyloid plaques, and it has 42 amino acids. First, we allow lyophilized Aβ_1-42_ to equilibrate at room temperature for 30 min to avoid condensation upon opening the peptide vial. Under the fume hood, re-suspend Aβ_1-42_ peptide in ice-cold HFIP to obtain a 1 mM solution and vortex the solution for a few seconds. Using a glass GasTight Hamilton syringe with Teflon plug, quickly divide the Aβ_1-42_/HFIP solution equally into three polypropylene vials and seal the vials. It was dissolved to a concentration of 100 μg/μl in 100% DMSO and kept at 4°C. Then it was resuspended in 100% DMSO, dissolved to a concentration of 100 μg/μl, and transferred to 4°C for 24 h to prepare an oligomer for use.

Aβ_1-42_ is the component found in amyloid plaques, and it has 42 amino acids. We used gradient concentrations of Aβ_1-42_ (0, 2.215, 4.43, 8.86, 17.72 μM) induced PC12 cell for 6, 12, 24, and 48 h (Neta et al., 2019). Cellular viability and toxicity were detected by CCK8 and LDH assays.

For cell growth and pharmacodynamics analyses, the cells were divided into six groups: normal group (PC12 cells), model group (culture with 7 μM Aβ_1-42_), β-asarone 24, 36, 72 μM, and donepezil 9.6 μM groups. For autophagy analyses, the cells were divided into six groups: normal group (PC12 cells); model group (cultured with 7 μM Aβ_1-42_); β-asarone 36 μM, A3 1 μM, CSA 20 μM, and 3MA 200 μM.

### Cell Proliferation and Cytotoxicity

Cell viability was detected using the CCK-8 assay. For the CCK-8 assay, PC12 cells were seeded in 96-well plates and cultured for 24 h. The absorbances were measured at 450 nm. Each group of experiments included six replicates and was repeated three times.

Cell damage was assessed by measuring LDH activity in PC12 cell supernatants using the LDH kit according to the manufacturer's protocol. In brief, 10 μl lysis buffer was added to high control at 24 h after drug treatment and incubated for 30 min at room temperature. Subsequently, 100 μl of working solution was added to each well. The plate was protected from light and incubated at 37°C for 30 min.

### Cellular Senescence Detection

PC12 cells were treated as the above groups. The culture medium was discarded, and the cells were washed with 2 ml HBSS once. Bafilomycin A1 working solution and SPiDER-βGal 1 ml were added in sequence to the culture for 1 h and 30 min, respectively. Then, the cells were washed with HBSS once again. All the data were observed under a fluorescence microscope.

### Immunofluorescence Analysis

We examined the expression of BACE1, APP, PS1, SYN1, LC3, BECN, and P62 with immunofluorescence (IF). PC12 cells were treated by grouping. Then, endogenous peroxidase activity was quenched for 10 min in PBS containing 3% H_2_O_2_, and the cells were chilled in water, and then immersed for 5 min in PBS. Sections were then incubated in anti-rabbit BACE1 (diluted 1:200), APP (diluted 1:100), PS1 (diluted 1:100), SYN1 (diluted 2:100), LC3 (diluted 1:100), BECN (diluted 1:100), and P62 (diluted 1:100) for 60 min at 37°C. Then the slides were set with SABC-FITC (diluted 1:200) and DAPI (1:5,000) for 5 min. Next, the cells were mounted with an anti-fluorescent quencher and blocked, and the specimen was visualized using a fluorescence microscope.

All the data were analyzed with ImageJ with integrated density. Each column in the results table calculates and displays the mean, standard deviation, minimum, and maximum of the values in that column.

### Western Blot Analysis

The AD pathology-related proteins BACE1, APP, PS1, Aβ, and SYN1 and the autophagy/mitophagy-related proteins LC3, BECN, P62, PINK1, and Parkin were also detected with WB. The cells were cultured in six-well plates for the above six groups. Protein samples were extracted from the cells by suspension in radioimmunoprecipitation assay (RIPA) buffer. Then the protein concentrations were determined using the Bradford protein method and the BCA protein assay kit. The membranes were washed twice, blocked for 10 min, and incubated for 24 h with antibodies to detect BACE1 (1:1,000), APPL (1:1,000), PS1 (1:1,000), Aβ (1:1,000), SYN1 (1:1,000), LC3 (1:400), BECN1 (1:2,000), PINK1 (1:1,000), Parkin (1:1,000), and actin (1:5,000). Then followed by horseradish peroxidase (HRP)–conjugated secondary antibodies (1:2,000). The membranes were washed three times, and then visualized by enhanced chemiluminescence kit prior to analysis with photographic imaging equipment. All the data were analyzed with Image lab; all bands were compared with actin first.

### Statistical Analysis

All data were expressed as the means ± standard deviation. Differences between two groups were analyzed using the t-test, and differences among three or more groups were analyzed using single-factor analysis of variance (one-way ANOVA) with SPSS. Differences were considered statistically significant at *P* < 0.05.

Since two sets of variables existed and the outcome set included more than one variable, the Canonical Correlation Analysis (CCA) method was performed (RStudio). We used the IF results of APP, PS1, BACE1, and SYN1 compared to BECN, P62, and LC3. The results and coefficients of the correlation analysis are expressed in heat maps.

## Results

### Aβ_1-42_ Induced PC12 Cell Model of AD in Gradient Concentrations and Times

**Figure 1:**
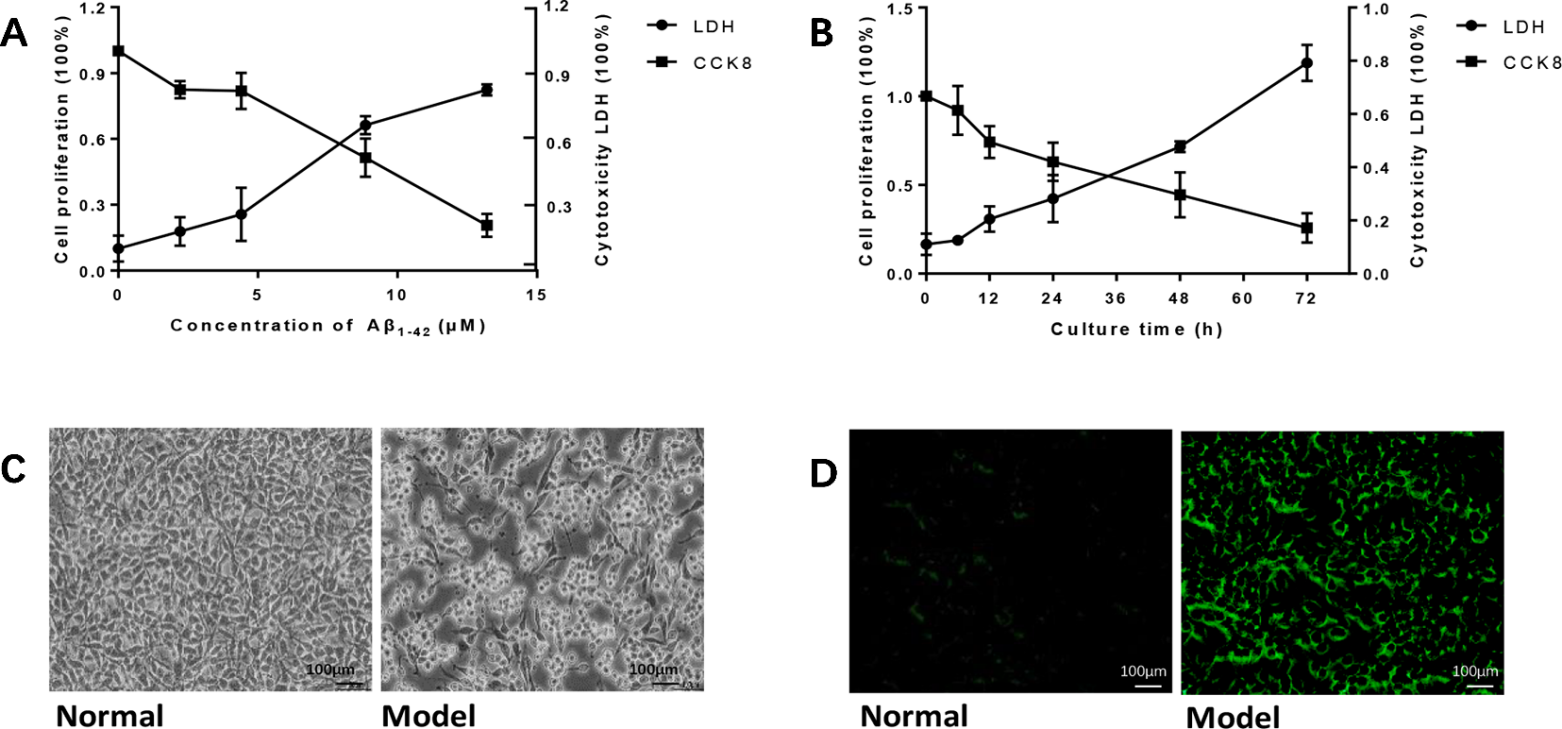
Amyloid β_1-42_ (Aβ_1-42_) induced PC12 cell model of Alzheimer's disease (AD) in gradient concentrations and times. **(A)** PC12 cells were cultured with Aβ_1-42_ 0, 2.215, 4.43, 8.86, 17.72 μM; Aβ_1-42_ decreased cell viability and increase cytotoxicity in a dose-dependent manner; 1/2 LD_50_ was 7 μM (Quest Graph™ LD_50_ Calculator. **(B)** PC12 cells were cultured with 7 μM Aβ_1-42_ for 6, 12, 24, 48, and 72 h; 12h was the best as 1/2 LD_50._ **(C)** Cell morphology was also clearly changed while the cells were cultured by Aβ_1-42_ for 12 h (×150), scale bar 100 μm; the appearances of cells were shrinking and flat, and protuberances disappeared. **(D)** Cellular senescence (green) was observed by fluorescence microscopy (excitation: 488 nm, emission: 500–600 nm), scale bar 100 μm. Senescent cells also increased in model group. The experiments were repeated twice independently with similar results.

To establish the AD cell model, we cultured PC12 cells *in vitro*, and Aβ_1-42_ was added into the medium in different concentrations and time points. As the concentration of Aβ_1-42_ and time increased, PC12 cell viability decreased in a dose-dependent manner; at the same time, cytotoxicity LDH increased (**Figures 1A, B**). We calculated LD50 with the Quest Graph™ LD50 Calculator (MLA, 2019) and chose 7 μM at 12 h as the experiment condition. The normal PC12 cells have a clear outline, and the shape is mostly round, spindle-shaped, and elliptical. The cells were also clustered while the dendrites were clearly visible. While the cells were damaged by 7 μM Aβ_1-42_ for 12 h, the appearances of cells were shrinking and flat, and protuberances disappeared (**Figure 1C**). Moreover, senescent cells also clearly increased in cells treated with Aβ_1-42_ (**Figure 1D**, n = 6).

### B-Asarone Inhibited Cell Damage in the Aβ_1-42_ PC12 Cell Model of AD

**Figure 2:**
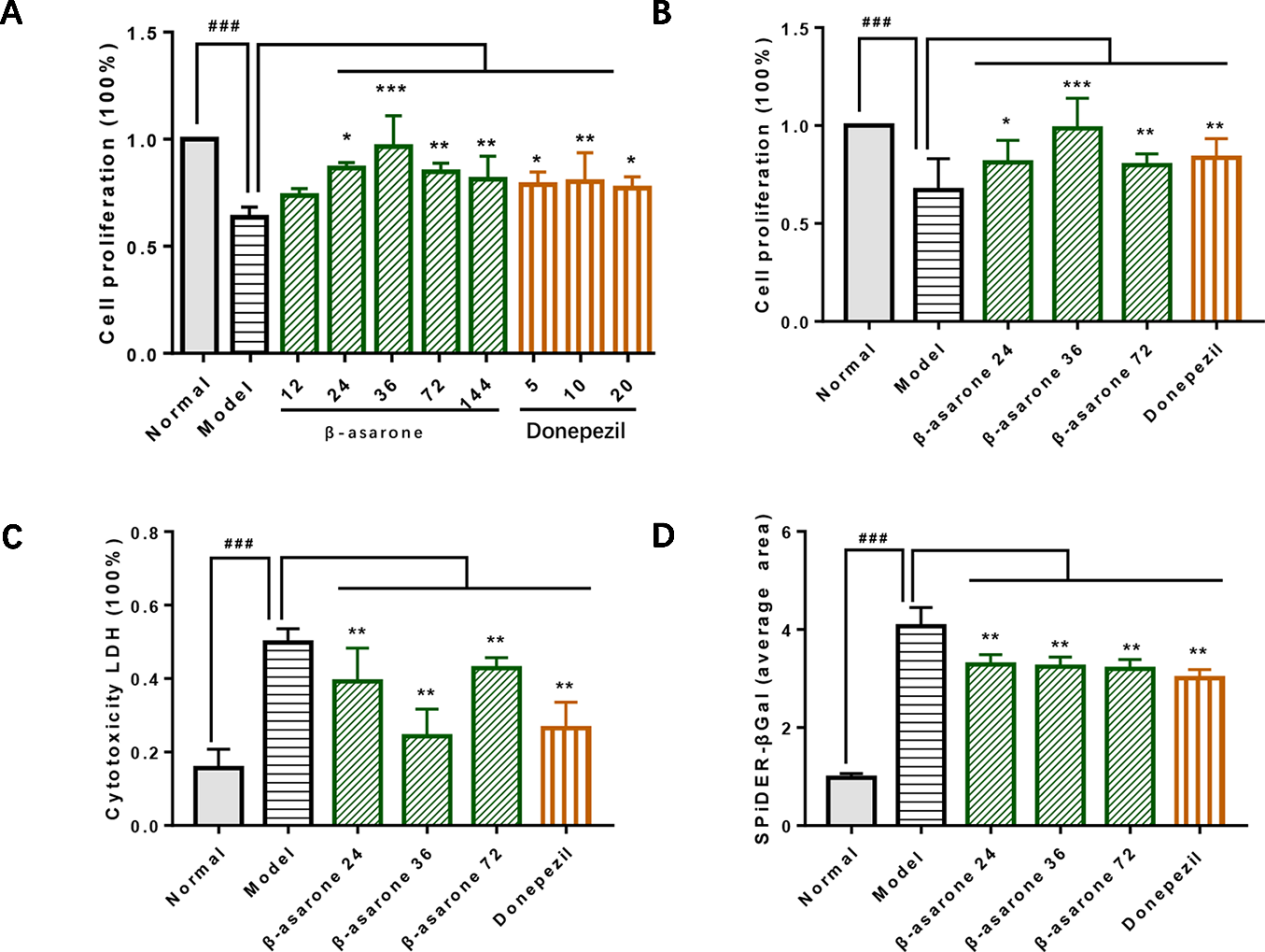
β-Asarone inhibited cell damage in Aβ_1-42_ PC12 cell model of AD. **(A)** PC12 cells were treated with 7 μM Aβ_1-42_ for 12 h; then we cultured cells with β-asarone 12, 24, 36, 72, 144 μM or donepezil 10, 20, 40 μM for another 12 h. Cell proliferation was examined with CCK8; β-asarone 24, 36, and 72 μM and donepezil 9.6 μM were the optimal dosage in inhibiting cell damage. **(B)** After culturing with the above group dosage, CCK8 analysis showed that cell proliferation was improved in β-asarone 24, 36, and 72 μM and donepezil 9.6 μM. **(C)** Cytotoxicity LDH was also detected; cell damage was improved in β-asarone 24, 36, and 72 μM and donepezil 9.6 μM. **(D)** The average area of SPiDER-βGal was examined with ImageJ; the senescent cells were all mitigated significantly compared with model cells. (Compared with normal group, ^#^ *P* < 0.05, ^##^ *P* < 0.01, ^###^ *P* < 0.001; compared with model group, *P < 0.05, **P < 0.01, ***P < 0.001; one-away ANOVA, n = 9).

After we established a stable AD cell model, we investigated the effects of gradient concentrations of β-asarone (12, 24, 36, 72, 144 μM) or donepezil (10, 20, 40 μM). We found that the protective effect of β-asarone on cell proliferation was dose-dependent; the low-dose group showed better protective effect than the high-dose group. PC12 cells grew dose-dependent in 12 to 36 μM; when the concentration was increased to 72 μM, the proliferation capacity of PC12 cells started to decrease (**Figure 2A**). We chose 24, 36, and 72 μM of β-asarone and 9.6 μM of donepezil as the working concentration, respectively. Compared with model cells, β-asarone and donepezil both improved cell proliferation and decreased cell damage (**Figures 2B, C**). At the same time, they also decreased the cell senescence rate (**Figure 2D**).

### β-Asarone Suppressed the Expression of APP, PS1, Aβ, and BACE1 While Promoting SYN1

**Figure 3:**
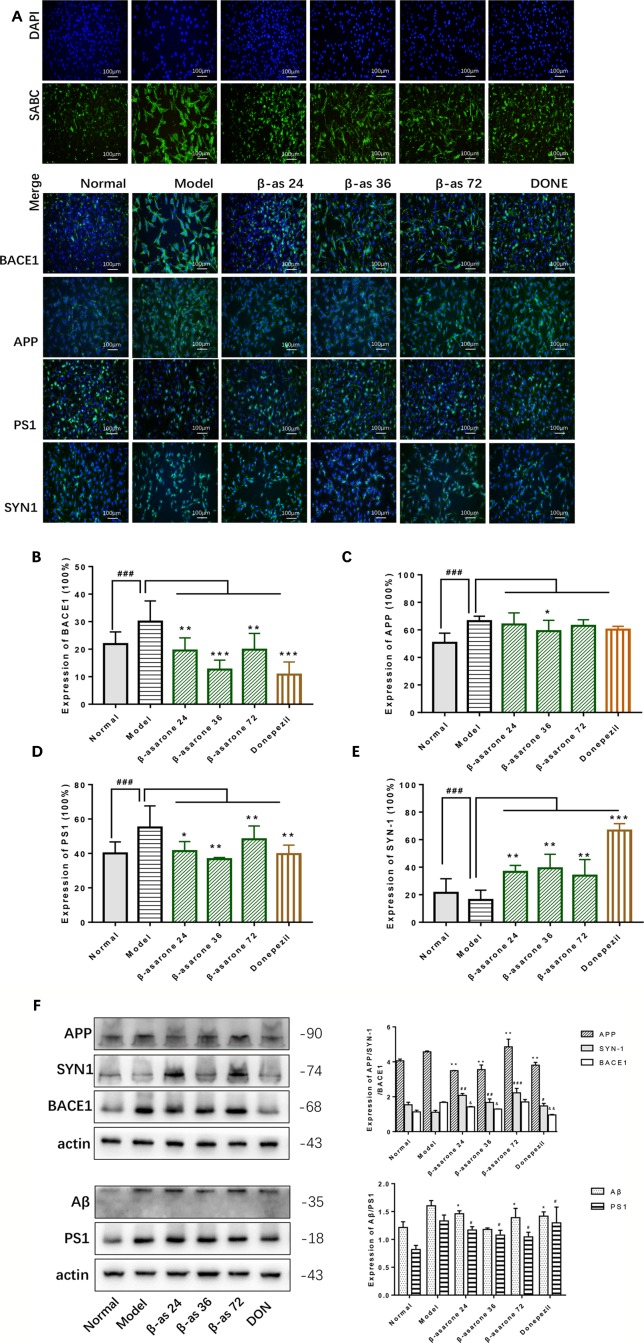
β-Asarone induction ameliorated Aβ pathology and synaptic damage in Aβ_1-42_ PC12 cell model of AD. PC12 cells were treated with 7 μM Aβ_1-42_ for 12 h; then we cultured the model cells with β-asarone 24, 36, 72 μM or donepezil 9.6 μM for another 12 h. **(A)** IF microscopy and data analysis of BACE1 **(B)**, APP **(C)**, PS1 **(D)**, and SYN-1 **(E)** showed: compared with normal group, APP, PS1, and BACE1 all increased significantly. β-asarone could decrease the expression of BACE1, APP, and PS1; donepezil was effective in reducing the expression of BACE1 and PS1. SYN-1 were all improved after culture with them. (Compared with normal group, ^#^ *P* < 0.05, ^##^ *P* < 0.01, ^###^ *P* < 0.001; compared with model group, *P < 0.05, **P < 0.01, ***P < 0.001; one-away ANOVA, n = 6). **(F)** Western blot data showed the effects of β-asarone on the expression levels of proteins involved, APP, SYN-1, BACE1, Aβ, and PS1. β-asarone suppressed the expression of APP, PS1, Aβ, and BACE1 and promoted SYN1. (Compared with model group, *P < 0.05, **P < 0.01, ***P < 0.001; one-away ANOVA, n = 6).

Overexpression of Aβ plaques deposition is the major neuropathological feature of AD. Aβ generation is initiated by the proteolysis of APP by BACE1. Synaptophysin (SYN) is a major integral membrane glycoprotein of neuronal synaptic vesicles, is present in virtually all synapses, and shows a high degree of evolutionary conservation across mammals. To investigate the protective role of β-asarone against AD pathology, we monitored the effects of β-asarone and donepezil in cell model with IF (**Figures 3A, F**) and Western blotting. β-Asarone suppressed the expression of BACE1 (**Figure 3B**), APP (**Figure 3C**), PS1 (**Figure 3D**), and Aβ (**Figure 3F**), while it promoted SYN1 (**Figure 3E**).

### β-Asarone Promoted the Expression of LC3 I/II, BECN, PINK1, and Parkin While It Inhibited P62

**Figure 4:**
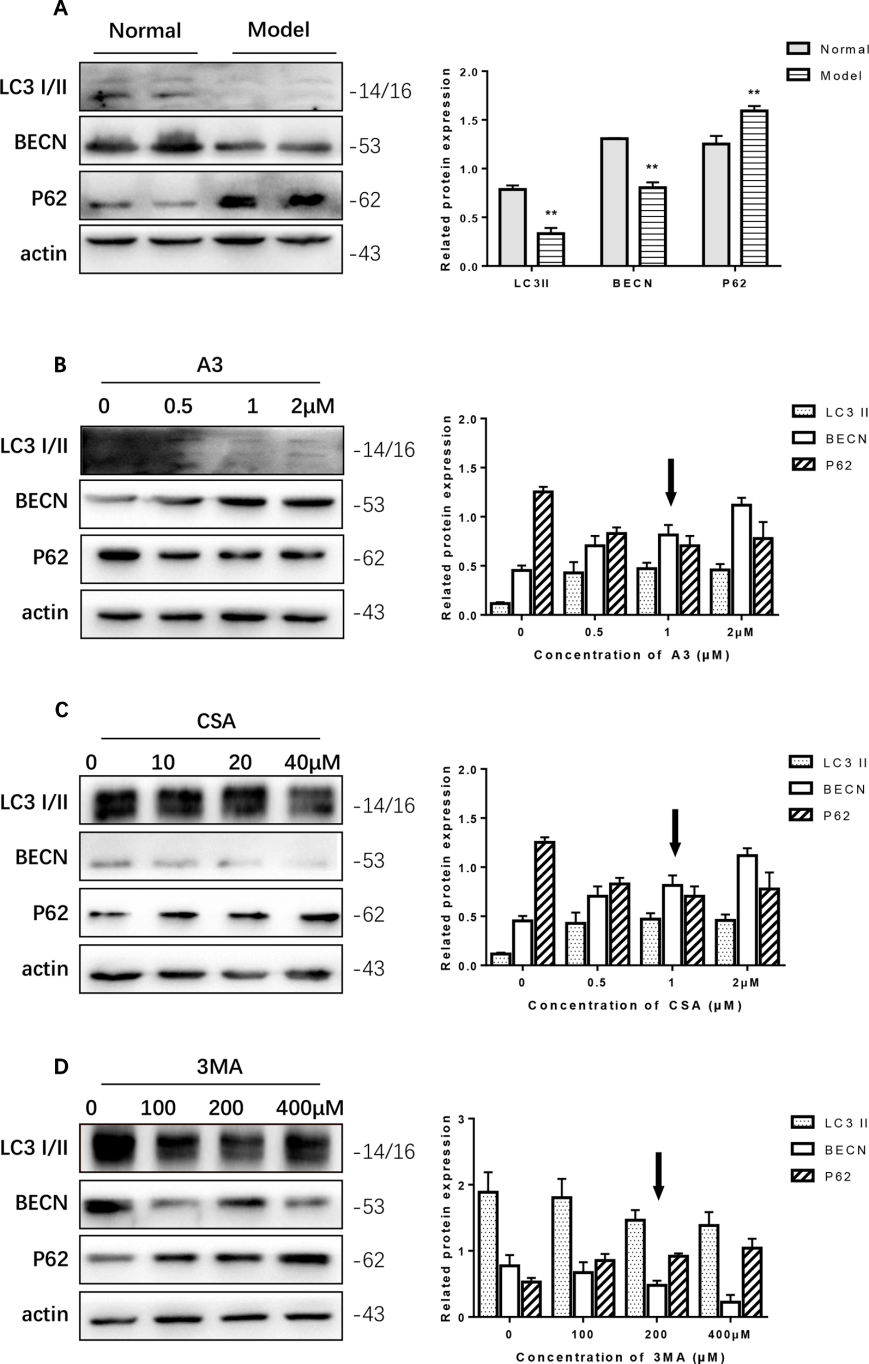
Optimum concentration of antimycin A (A3), cyclosporine A (CSA), and 3-methyladenine (3MA) for Aβ_1-42_ PC12 cell model of AD. **(A)** Autophagy-related protein changed in AD cell model. Compared with normal pc12 cells, the expression of LC3 I/II and Beclin-1 increased while p62 decreased after culturing with 7 μM Aβ_1-42_ for 12 h. (Compare with the normal group, **P < 0.01). AD cells were treated with **(B)** A3 0.5, 1.2 μM, **(C)** CSA 10, 20, 40 μM, **(D)** 3MA 100, 200, 400 μM for 12 h. Then the autophagy-related proteins were examined with Western blot. The optimum concentration of them were as follows: A3 1 μM, CSA 20 μM, and 3MA 200 μM respectively.

In the PC12 cell AD model, we found that autophagy was inhibited. The Western blot expression levels of LC3 I/II and BECN were decreased while P62 showed the opposite effect (**Figure 4A**). We used A3 as an autophagy activator and CSA and 3MA as autophagy inhibitors. Hence, to choose the optimal dosage, we detected them in gradient concentrations and analyzed the data. The final concentrations we selected of A3 (**Figure 4B**), CSA (**Figure 4C**), and 3MA (**Figure 4D**) were 1, 20, and 200 μM, respectively.

**Figure 5:**
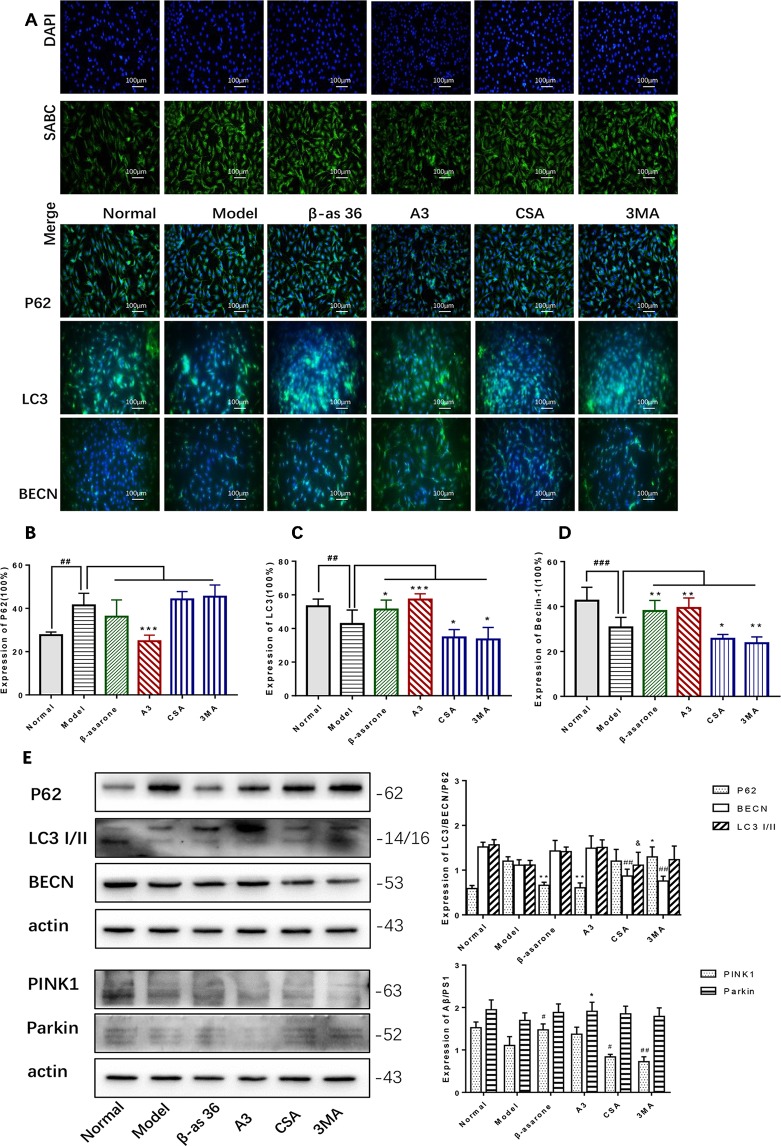
β-asarone promoted autophagy- and mitophagy-related proteins in Aβ_1-42_ PC12 cell model of AD. PC12 cell was treated with 7 μM Aβ_1-42_ for 12 h; then we cultured the model cells with β-asarone 36 μM, A3 1 μM, CSA 20 μM, and 3MA 200 μM respectively for another 12 h. **(A)** Immunofluorescence (IF) microscopy and data analysis of P62 **(B)**, LC3 **(C)**, and BECN **(D)** showed: compared with normal group, P62 increased while LC3 and BECN decreased; β-asarone could improve the expression of LC3 and BECN. (Compared with normal group, ^#^ *P* < 0.05, ^##^ *P* < 0.01, ^###^ *P* < 0.001; compared with model group, *P < 0.05, **P < 0.01; one-away ANOVA, n = 6). **(E)** Western blot data showed the effects of β-asarone on the expression levels of proteins involved P62, LC3, BECN, PINK1, and Parkin. β-asarone showed effect on reducing P62 and improving PINK1. (Compared with model group, ^#^ *P* < 0.05, ^##^ *P* < 0.01; *P < 0.05, **P < 0.01; ^&^ *P* < 0.05; one-away ANOVA, n = 6).

PINK1 and Parkin were shown to mediate the degradation of damaged mitochondria *via* selective autophagy (mitophagy). We next examined whether β-asarone could affect autophagy and mitophagy function with IF and Western blot. Clearly we could declare from the IF result (**Figure 5A**) that β-asarone could promote autophagy *via* an increase in the expression of LC3 I/II and BECN (**Figures 5C, D**). But p62 has little change (**Figure 5B**). Moreover, we preliminary found β-asarone could induce PINK1 (**Figure 5E**). Because autophagy failure was supposed to be the major driver of cell death in AD, our results suggested that β-asarone could protect AD cells by promoting autophagy in a model of AD.

### Autophagy Defects Have a Critical Role in the Formation of the AD Pathological State

**Figure 6:**
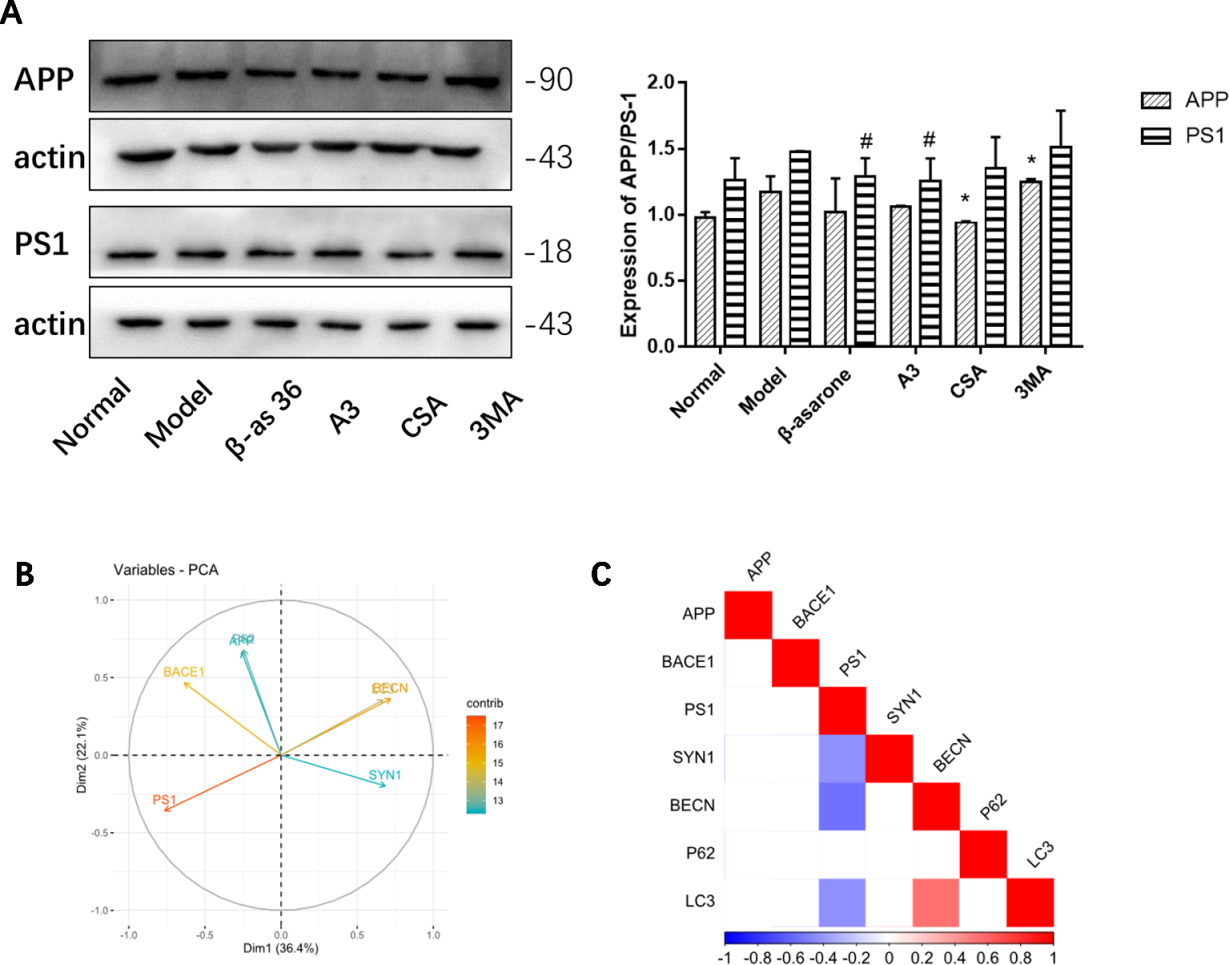
The relationships of AD and autophagy factors. **(A)** Representative Western blotting bands using total protein extracts from the cells. Compared with model group, β-asarone and A3 both decreased the expression of PS1. β-asarone can promote the occurrence of autophagy and reduce PS1. (Compare with normal group ,^#^p < 0.05; compare with model group, *p < 0.05; one-away ANOVA, n=6). **(B)** The results of Canonical Correlation Analysis (CCA) showed a highly significant relationship between the pathology factor of AD and protein expression of autophagy. **(C)** *P* < 0.05 was not shown; basically P62 was negatively correlated with BECN (r = −0.68, *P* = 0.149) and LC3 (r = −0.119, *P* = 0.06); BECN and LC3 were positively related (r^2^ = 0.547, *P* = 0.006). PS1 was negatively correlated with BECN1 (r^2^ = −0.556, *P* = 0.005) and LC3 (r^2^ = −0.430, *P* = 0.040). Furthermore, PS1 was also negatively correlated with SYN1 (r^2^ = −0.424, *P* = 0.041) (Pearson correlation coefficient, n = 28).

Compared with model group, β-asarone and A3 both decreased the expression of PS1. β-Asarone can promote the occurrence of autophagy and reduce PS1 (**Figure 6A**). This also gives us some hints that β-asarone can promote the occurrence of autophagy; at the same time, it can reduce the expression of AD-related proteins. To further prove this result, we did a correlation analysis. The results of CCA showed a highly significant relationship between the pathological factors of AD and the autophagy proteins expressed (**Figure 6B**). Basically, of the core proteins in autophagy that we chose, P62 was negatively correlated with BECN and LC3. PS1 was negative with BECN and LC3, which suggests that the increase in PS1 is most likely due to the destruction of autophagy. On the other hand, SYN1 was also negatively correlated with PS1 (**Figure 6C**).

## Discussion

AD is one of the most common causes of mental deterioration in elderly adults, accounting for approximately 50%–60% of the total dementia in people over 65 years of age (Park et al., 2019). In the past two decades, researchers have made great efforts to explore and discover the cause of AD, and ultimately hope to develop safe and effective therapeutic drugs (Pansieri et al., 2019). There are different hypotheses about the onset of the disease so far; the clinical drugs developed for AD are all based on protecting cholinergic and non-cholinergic neurons from the neurochemical and histopathological changes that occur in AD. Currently, there are no treatments available to alleviate AD or control its progression. The discovery and development of cholinesterase inhibitors of AD is recognized as one of the most effective treatments to improve mood changes and reduce movement disorders. However, clinical follow-up studies have found that these treatments do not effectively prevent AD progression, and do not perform well in improving patients' memory impairment and daily life.

Aβ fulfills a central role in AD pathogenesis, and reduction in Aβ levels should prove beneficial for AD treatment. The generation of Aβ is initiated by the proteolysis of APP by BACE1 (Cole and Vassar, 2008). As such, many institutes have been experimenting with blocking this production by producing BACE1 inhibitors. However, over the years, these have proven unsuccessful for a variety of reasons. Several trials of BACE1 inhibitors have been suspended due to their inability to improve AD symptoms or because of adverse side effects, despite reducing Aβ plaque formation. Many scientists think that BACE inhibitors fail as they prevent amyloid production later in the course of illness and may be more effective if used earlier. Trials failing on safety issues may be due to the structure of the drug (MLA, 2018).

As a commonly used herb belonging to the Acoraceae family, ATS has been reported in the literature for its effects on nervous system diseases such as neurodegenerative diseases, hypoxic-ischemic encephalopathy, and cerebrovascular diseases (Zhu et al., 2014). β-Asarone is the main effective component of ATS volatile oil. The molecular mass of β-asarone is 208. It is fat soluble and can easily pass the BBB and distribute mainly into the brain (Ning et al., 2019).

In this study, we focused on the pharmacological activities of β-asarone in a stable AD cell model. Groups included gradient concentrations of β-asarone and donepezil. Donepezil hydrochloride is the only new drug approved by both the FDA and MCA for the symptomatic treatment of mild and moderate AD, so we chose it as positive control drug (Petersen et al., 2005). The cell proliferation and LDH experiments showed that both β-asarone and donepezil could improve the growth of model cells. Furthermore, it could also affect BACE1, and whether this could be an effective BACE inhibitor needs to be further verified with experimentation.

Based on the above results, we confirmed that this mechanism is the basis of the protective effect of β-asarone in AD. We also demonstrated that it has a significant therapeutic effect against toxic protein deposition. At the same time, β-asarone could also improve the expression of SYN1, which should destroy the superoxide anion radicals produced in cells that are toxic to biological systems. So, it plays a dual role of inhibition and promoting binding by eliminating metabolic toxicants and nutritional synapses.

Here we used LC3 I/II, BECN, P62, PINK1, and Parkin as indicators to detect autophagy. As the most important marker of autophagy, LC3 is now widely used to monitor autophagy. Thus, the detection of LC3 conversion (LC3-I to LC3-II) analysis is more correct because the amount of LC3-II is related to the number of autophagosomes (Mizushima and Yoshimori, 2007). BECN was the first mammalian gene which plays a role in mediating autophagy (Sun and Peng, 2008). P62 is a selective substrate for autophagy and delivers aggregates for autophagy degradation *via* LC3 interaction regions (Johansen and Lamark, 2011). Recently, PINK1 and Parkin have been implicated in the same signaling pathway to regulate mitochondrial clearance through the recruitment of Parkin by the stabilization of PINK1 on the outer membrane of depolarized mitochondria (Lazarou et al., 2015). The PINK1-dependent pathway also eliminates proinflammatory cytokines in AD microglia (Ye et al., 2015).

Our study's statistical significance and effect sizes demonstrate that there is a remarkable relationship between AD and autophagy. These findings provide evidence that autophagy is suppressed in an AD cell model, and that β-asarone plays a critical role in restoring autophagy. More broadly, we also detected the expression of PINK1 and Parkin; β-asarone can also affect mitophagy and improve energy metabolism.

However, it also raises many important questions, such as: (1) Does β-asarone only work by clearing the misfolded protein? (2) AD is a progressively aggravating disease. When is the best time for the application of β-asarone? While it is difficult to address these questions with cell models, we are conducting related animal experiments to further explain this problem.

## Conclusion

In conclusion, our analyses provided evidence that autophagy defects have a critical role in AD development and progression. β-Asarone could protect the PC12 cell model against the formation and damage of Aβ_1_-_42_, and this process should occur by promoting autophagy. Therefore, targeting autophagy at the neuronal and biological levels may be a promising therapeutic approach.

## Data Availability Statement

All datasets generated for this study are included in the article/**Supplementary Material**.

## Ethics Statement

All the experiments complied with ARRIVE guidelines and were carried out in strict accordance with the recommendations in the Guide for the Care and Use of Laboratory Cells of Jinan University.

## Author Contributions

Study design: RZ and NW. Study conduct: NW, HW, and YL. Data collection: HW, LL, and YL. Data analysis: NW and HW. Data interpretation: NW. Drafting manuscript: NW, HW, YL, and LL. Approving the final version of the manuscript: all authors.

## Funding

This work was supported by the National Natural Science Foundation of China (81903971), General Program of China Postdoctoral Science Foundation (2018M643377), and National Key R&D Program of China (2018YFC2002500).

## Conflict of Interest

The authors declare that the research was conducted in the absence of any commercial or financial relationships that could be construed as a potential conflict of interest.
