# Supplementary material for: β-Asarone Inhibits Amyloid-β by Promoting Autophagy in a Cell Model of Alzheimer's Disease
Source: Front Pharmacol. 2020 Jan 17;10:1529. doi: 10.3389/fphar.2019.01529 (PMC6979317; doi:10.3389/fphar.2019.01529)
Supplement: Supplementary file 1 [file DataSheet_1.pdf]

β-细辛醚

beta-Asarone

【类别】化学对照品

【批号】112018-201601

【结构式】

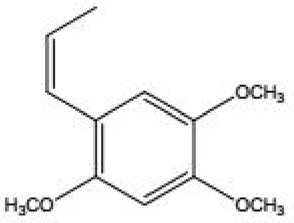

【分子式】$C_{12}H_{16}O_3$

【分子量】208.11

【CAS 号】5273-86-9

【用途】β-细辛醚（beta-Asarone）对照品系供中国药典 2015 版药典癫痫平片项下含量测定用。

【特性量值】含量以 96.8%计。

【包装】棕色安瓿

【规格】20mg/支

【贮藏】冷冻保存。

【使用方法】使用前不需干燥处理。

【有效期】国家药品标准物质不设具体有效期，按照规定条件保存的标准物质，在中国食品药品检定研究院发布停用通知前有效。

声明：

1.请按本品说明书规定使用，若作他用，用户须自行证明适用性；

2.因用户使用或储存不当所引起的损害或投诉,用户自行承担相关责任；

3.用户收到本品后应立即核对品种、数量、包装等，若出现质量、数量等不符，相关赔偿只限于标准物质本身，不涉及其他任何损失。
